# Comprehensive analysis of Pan-Immune Inflammation and all-cause mortality in rheumatoid arthritis: a database-driven approach, 1999-2018

**DOI:** 10.3389/fimmu.2025.1549955

**Published:** 2025-01-28

**Authors:** Muradil· Mardan, Huoliang Zheng, Qingyin Xu, Shaokuan Song, Zeyu Lu, Hui Deng, Hao Cai, Qizhu Chen, Bingyi Yang, Kudelaiti· Abuduwufuer, Pengbo Chen, Bo Li, Shengdan Jiang, Leisheng Jiang, Xin-feng Zheng

**Affiliations:** ^1^ Spine Center, Xinhua Hospital Affiliated to Shanghai Jiaotong University School of Medicine, Shanghai, China; ^2^ Department of Oncology, Shanghai Traditional Chinese Medicine Hospital, Shanghai, China

**Keywords:** rheumatoid arthritis, Pan-Immune Inflammation Value, all-cause mortality, systemic inflammation, prognostic biomarker, NHANES, Cox proportional hazards model, segmented regression analysis

## Abstract

**Background:**

Rheumatoid arthritis (RA) is a chronic autoimmune disease marked by systemic inflammation and immune dysregulation, leading to a higher risk of all-cause mortality. The Pan-Immune Inflammation Value (PIV), a novel biomarker capturing immune-inflammatory activity, has shown prognostic value in various diseases. However, its role in predicting outcomes in RA patients remains largely unexplored.

**Objectives:**

This study aimed to evaluate the association between PIV and all-cause mortality in RA patients, investigate nonlinear relationships, and identify threshold effects.

**Methods:**

Data from the 1999–2018 National Health and Nutrition Examination Survey (NHANES) were used, including 1,882 RA patients. PIV was calculated as (neutrophil count×platelet count×monocyte count)/lymphocyte count and categorized into quartiles (Q1–Q4). Multivariable Cox proportional hazards models were applied to assess the relationship between PIV and mortality, with results expressed as hazard ratios (HRs) and 95% confidence intervals (CIs). Restricted cubic splines (RCS) explored nonlinear trends, and segmented Cox regression identified threshold effects. Kaplan-Meier survival curves and subgroup analyses validated the findings and assessed potential modifiers.

**Results:**

Elevated PIV levels were strongly associated with increased all-cause mortality. Compared to Q1, adjusted HRs for Q2, Q3, and Q4 were 1.60 (95% CI: 1.01–2.53, P = 0.047), 1.70 (95% CI: 1.10–2.63, P = 0.016), and 2.12 (95% CI: 1.33–3.37, P = 0.002), respectively (P for trend < 0.001). RCS analysis revealed a nonlinear relationship with a threshold at PIV = 302. Below this threshold, increasing PIV was associated with higher mortality risk (HR = 1.67, 95% CI: 1.07–2.61, P = 0.024). Conversely, above the threshold, further increases in PIV were linked to reduced mortality risk (HR = 0.98, 95% CI: 0.97–0.99, P = 0.026). Kaplan-Meier survival curves showed a clear decline in survival probability with increasing PIV quartiles (P < 0.001). Subgroup analyses confirmed consistent findings, with a notable interaction observed in diabetic patients (P for interaction = 0.002).

**Conclusions:**

PIV is a significant and independent predictor of all-cause mortality in RA patients, characterized by a nonlinear association and a distinct threshold effect. These findings highlight the potential of PIV as a pragmatic biomarker for stratifying mortality risk and informing personalized treatment strategies in RA.

## 1 Introduction

Rheumatoid arthritis (RA) is a chronic autoimmune disorder characterized by persistent joint inflammation, progressive damage, and functional impairment, often leading to significant disability (1–3). Beyond its joint-related symptoms, RA increases the risk of systemic complications, such as cardiovascular disease and infections, which substantially elevate all-cause mortality (4). Chronic inflammation and immune dysregulation are pivotal drivers of these outcomes, highlighting the urgent need for effective biomarkers to improve prognosis and treatment strategies.

Various composite biomarkers have been utilized to evaluate disease activity and prognosis in rheumatoid arthritis patients (5). The Pan-Immune Inflammation Value (PIV) is an emerging composite biomarker derived from neutrophil, monocyte, platelet, and lymphocyte counts. It provides a comprehensive measure of immune and inflammatory activity (6–9). While PIV has been associated with poor outcomes in conditions like cancer and cardiovascular disease, its role in predicting mortality in RA patients remains unclear and largely unexplored (10–12).

This study leveraged data from the National Health and Nutrition Examination Survey (NHANES), a large, nationally representative cohort with rigorous methodology and extensive follow-up. Using this robust dataset, we investigated the association between PIV and all-cause mortality in RA patients.

We applied multivariable Cox proportional hazards models to analyze the relationship between PIV and mortality risk. Segmented regression models were used to identify potential thresholds in this association, while subgroup analyses assessed whether factors like demographics, lifestyle behaviors, or comorbidities influenced these findings.

By evaluating the prognostic value of PIV in RA patients, this study aims to highlight its potential as a mortality risk marker. The findings may support more personalized risk assessment and inform targeted strategies for inflammation management in RA.

## 2 Methods

### 2.1 Data source

This study utilized data from the NHANES, a large-scale survey conducted every two years in the United St ates since 1999. NHANES employs a multi-stage, stratified sampling method to ensure that participants are representative of the U.S. population across all age groups. Participants provided informed consent and completed questionnaires with assistance from trained staff, followed by physical examinations in mobile examination centers. Ethical approval for the survey was granted by the National Center for Health Statistics (NCHS). All-cause mortality data were obtained by linking NHANES records to the National Death Index (NDI), with follow-up data available through December 31, 2019. This publicly available data is accessible on the NHANES website (https://www.cdc.gov/nchs/nhanes/about_nhanes.htm).

**Figure 1:**
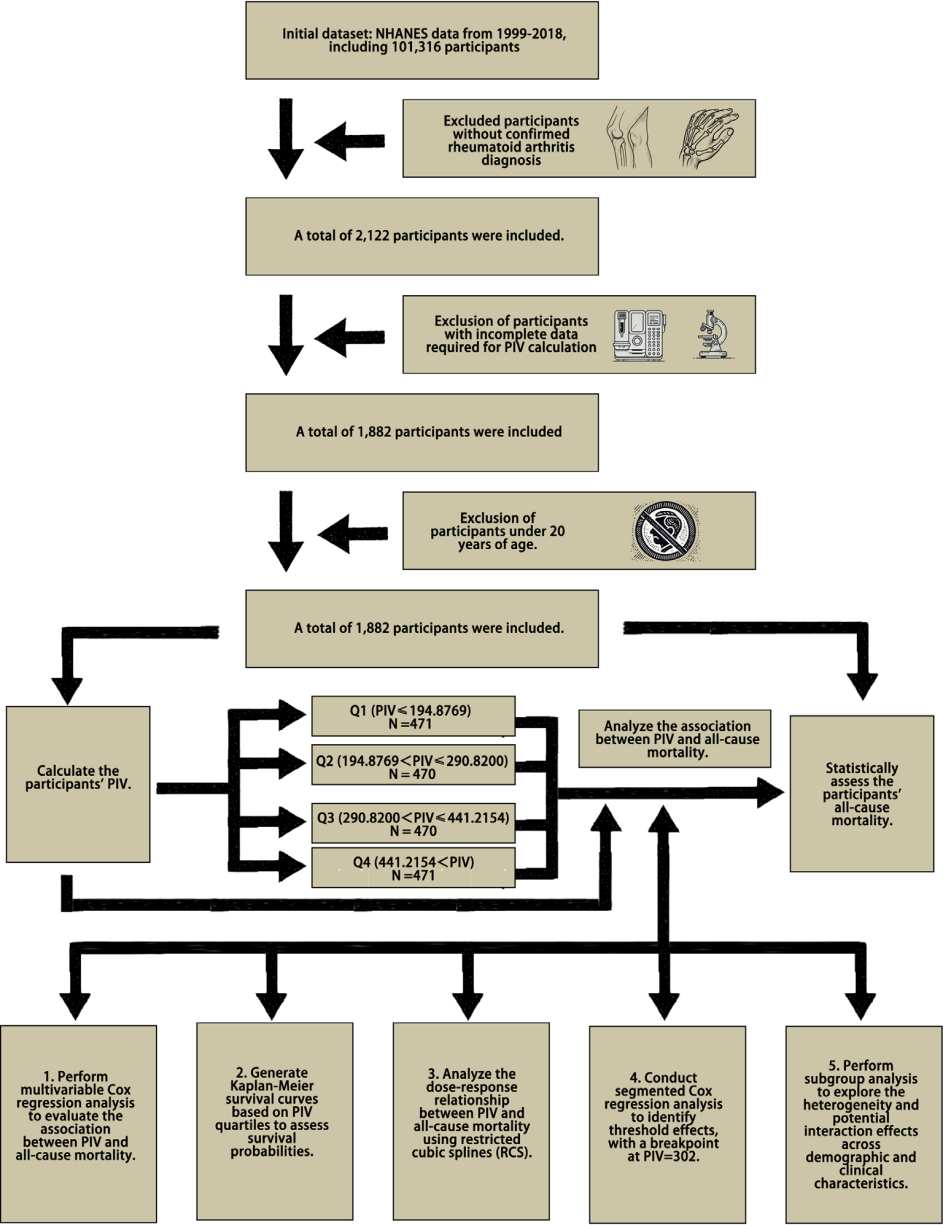
Flowchart of data selection and analysis for PIV and mortality.

From the initial pool of 101,316 participants in the 1999 to 2018 NHANES cycles, we excluded individuals without a confirmed diagnosis of RA and those missing key data required to calculate the PIV, derived from platelet, neutrophil, monocyte, and lymphocyte counts. Additionally, participants under the age of 20 were excluded. After applying these criteria, 1,882 participants were included in the final analysis (**Figure 1**).

### 2.2 Exposure and outcome variables

The PIV is calculated using the formula: PIV = (neutrophil count × platelet count × monocyte count)/lymphocyte count. Neutrophil, platelet, monocyte, and lymphocyte counts were obtained from laboratory blood tests. Based on the quartiles of PIV, the 1,882 participants were divided into four groups: Q1, Q2, Q3, and Q4. This study analyzed variations in different variables across these quartiles. While categorizing continuous variables into quartiles may lead to some loss of statistical power, this approach helps reduce bias, particularly when the variable distribution is skewed. Moreover, it allows for the exploration of potential relationships between PIV and mortality at different levels. Once PIV values were calculated, each participant was matched to all-cause mortality data from the National Death Index (NDI) for subsequent analyses.

### 2.3 Assessment of other covariates

Other covariates included demographic factors (age, sex, race, marital status, education level), lifestyle behaviors (smoking, drinking), and medical history (hypertension, diabetes). Anthropometric measures such as waist circumference, weight, height, and body mass index (BMI, kg/m²) were also considered. Smoking was defined as having smoked at least 100 cigarettes in one’s lifetime, while drinking was defined as consuming at least 12 alcoholic beverages within a single year, based on standardized questionnaires. Participants who confirmed being told by a doctor that they had diabetes or hypertension were classified accordingly. Blood samples were analyzed to measure total cholesterol (TC), triglycerides (TG), high-density lipoprotein cholesterol (HDL-C), and low-density lipoprotein cholesterol (LDL-C).

### 2.4 Statistical analysis

Continuous variables were expressed as means with standard deviations (SD) or medians with interquartile ranges (IQR), depending on their distribution, while categorical variables were summarized as frequencies and percentages (n, %). The normality of the data was assessed using the Kolmogorov-Smirnov test. Differences in normally distributed continuous variables were compared using one-way ANOVA, while the Wilcoxon-Mann-Whitney rank-sum test was used for non-normally distributed variables. Categorical variables were compared using Chi-square tests.

To analyze differences across PIV quartiles, appropriate statistical tests were applied based on the type and distribution of the variables. Multivariable Cox proportional hazards models were used to assess the association between PIV and all-cause mortality, with results presented as hazard ratios (HRs) and 95% confidence intervals (CIs). Restricted cubic splines were used to explore potential nonlinear relationships between PIV and mortality, and segmented Cox regression models were employed to identify threshold effects and breakpoints. The log-likelihood ratio test was used to compare the standard Cox model with the segmented model.

Subgroup analyses were conducted to evaluate whether demographic factors (e.g., age, sex), lifestyle behaviors (e.g., smoking, alcohol consumption), and clinical characteristics (e.g., hypertension, diabetes) modified the association between PIV and all-cause mortality. Interaction terms were included in the models to assess effect modification, and significant interactions were further analyzed to provide subgroup-specific insights.

All statistical analyses were performed using R software (version 4.2.3), with statistical significance set at a two-sided P < 0.05. Data management and additional analyses were carried out using IBM SPSS Statistics (version 25).

## 3 Results

### 3.1 Baseline characteristics

This study included 1,882 patients with RA, with an average age of 65.6 ± 13.6 years; 64.1% of participants were female, and 35.9% were male. Patients were divided into quartiles based on their PIV. Results indicated a significant increase in mortality rates with rising PIV levels, from 35.5% in the lowest PIV group (Q1) to 56.9% in the highest (Q4) (P < 0.001). Higher PIV levels were also closely associated with older age (P = 0.003) and a larger proportion of males (P = 0.046). Additionally, the high PIV group had a notably higher smoking rate (P < 0.001) and greater waist circumference (P = 0.026), reflecting poorer metabolic status. In this group, high-density lipoprotein cholesterol (HDL-C) was significantly lower (P = 0.003), while triglycerides were markedly higher (P = 0.036). These findings suggest that elevated PIV levels may be a marker for higher mortality risk in RA patients and are significantly associated with several adverse health characteristics (**Table 1**).

**Table 1:** Baseline characteristics of participants grouped by PIV levels: a cross-sectional analysis.

	Q1 (PIV≤194.8769) *N* =471	Q2 (194.8769<PIV ≤ 290.8200) *N* = 470	Q3 (290.8200<PIV ≤441.2154) *N* = 470	Q4 (441.2154<PIV) *N* =471	*P* Value
Gender, *n* (%)					0.046
Male	155 (32.9%)	162 (34.5%)	177 (37.7%)	181 (38.4%)	
Female	316 (67.1%)	308 (65.5%)	293 (62.3%)	290 (61.6%)	
Race/ethnicity, *n* (%)					0.019
Mexican American	47 (10.0%)	51 (10.9%)	46 (9.8%)	29 (6.2%)	
Other Hispanic	17 (3.6%)	17 (3.6%)	18 (3.8%)	12 (2.5%)	
Non-Hispanic White	280 (59.4%)	340 (72.3%)	359 (76.4%)	390 (82.8%)	
Non-Hispanic Black	108 (22.9%)	50 (10.6%)	35 (7.4%)	33 (7.0%)	
Other Race	19 (4.0%)	12 (2.6%)	12 (2.6%)	7 (1.5%)	
Education level, *n* (%)					0.446
Less Than 9th Grade	57 (12.1%)	52 (11.1%)	59 (12.6%)	43 (9.1%)	
9-11th Grade	71 (15.1%)	65 (13.8%)	67 (14.3%)	65 (13.8%)	
High School Grad/GED or Equivalent	111 (23.6%)	120 (25.5%)	115 (24.5%)	121 (25.7%)	
Some College or AA degree	124 (26.3%)	121 (25.7%)	122 (26.0%)	138 (29.3%)	
College Graduate or above	108 (22.9%)	112 (23.8%)	107 (22.8%)	104 (22.1%)	
Refused	0 (0%)	0 (0%)	0 (0%)	0 (0%)	
Don’t Know	0 (0%)	0 (0%)	0 (0%)	0 (0%)	
Marital Status, *n* (%)					0.178
Married	271 (57.5%)	276 (58.7%)	276 (58.7%)	254 (53.9%)	
Widowed	94 (20.0%)	86 (18.3%)	99 (21.1%)	104 (22.1%)	
Divorced	63 (13.4%)	59 (12.6%)	52 (11.1%)	53 (11.3%)	
Separated	13 (2.8%)	7 (1.5%)	6 (1.3%)	13 (2.8%)	
Never Married	22 (4.7%)	26 (5.5%)	21 (4.5%)	32 (6.8%)	
Living with Partner	8 (1.7%)	16 (3.4%)	16 (3.4%)	15 (3.2%)	
Refused	0 (0%)	0 (0%)	0 (0%)	0 (0%)	
Smoking, *n* (%)					<0.001
Yes	220 (46.7%)	242 (51.5%)	252 (53.6%)	281 (59.7%)	
No	251 (53.3%)	228 (48.5%)	218 (46.4%)	190 (40.3%)	
Drinking, *n* (%)					0.168
Yes	310 (65.8%)	337 (71.7%)	313 (66.6%)	297 (63.1%)	
No	161 (34.2%)	133 (28.3%)	157 (33.4%)	174 (36.9%)	
Diabetes mellitus, *n* (%)					0.671
Yes	79 (16.8%)	53 (11.3%)	92 (19.6%)	71 (15.1%)	
No	392 (83.2%)	417 (88.7%)	378 (80.4%)	400 (84.9%)	
Hypertension, *n* (%)					0.262
Yes	267 (56.7%)	249 (53.0%)	276 (58.7%)	276 (58.6%)	
No	204 (43.3%)	221 (47.0%)	194 (41.3%)	195 (41.4%)	
Age (yrs), mean (SD)	64.85 (12.22)	64.14 (13.56)	66.65 (13.89)	66.79 (14.64)	0.003
Weight (kg)	81.74 (19.30)	81.67 (20.04)	82.58 (22.95)	80.32 (20.55)	0.442
Standing Height (cm), mean (SD)	166.06 (9.69)	164.85 (10.15)	165.40 (10.11)	165.16 (10.32)	0.303
Body Mass Index (kg/m²), mean (SD)	29.56 (6.23)	29.92 (6.23)	30.00 (7.21)	29.42 (6.75)	0.805
Waist Circumference (cm), mean (SD)	100.59 (13.83)	101.20 (14.41)	102.79 (16.34)	102.42 (15.38)	0.026
LDL-cholesterol (mmol/L), mean (SD)	3.11 (0.99)	3.19 (0.99)	3.02 (0.89)	3.07 (0.90)	0.348
Direct HDL-Cholesterol (mmol/L), mean (SD)	1.48 (0.46)	1.44 (0.41)	1.41 (0.40)	1.39 (0.41)	0.003
Triglyceride (mmol/L), mean (SD)	1.68 (1.25)	1.67 (0.84)	1.83 (0.94)	1.85 (1.17)	0.036
Total Cholesterol (mmol/L), mean (SD)	5.33 (1.12)	5.32 (1.09)	5.29 (1.08)	5.31 (1.07)	0.780
Status, n (%)					<0.001
Alive	304 (64.5%)	277 (58.9%)	220 (46.8%)	203 (43.1%)	
Death	167 (35.5%)	192 (40.9%)	249 (53.0%)	268 (56.9%)	

### 3.2 Associations between PIV and mortality

As shown in **Table 2**, PIV was significantly associated with the risk of all-cause mortality in RA patients. In the crude model (Model 1), each unit increase in PIV was associated with a hazard ratio (HR) for all-cause mortality of 1.0006 (95% CI: 1.0005–1.0008, P < 0.0001). This association remained statistically significant and robust across different models. In Model 2, after adjusting for gender, race/ethnicity, education level, marital status, and age, the HR was 1.0003 (95% CI: 1.0002–1.0005, P < 0.0001). Further adjustments in Model 3, which included smoking, hypertension, diabetes, alcohol consumption, body measurements, and lipid levels, yielded an HR of 1.0004 (95% CI: 1.0001–1.0007, P = 0.0085).

**Table 2:** Impact of PIV levels on all-cause mortality in RA patients: stratified and non-stratified Cox proportional hazards regression analysis.

	Crude model (Model 1)		Minimally adjusted model (Mol 2)		Fully adjusted model (Model 3)	
	HR^*^(95% CI)	*P* value	HR^*^(95% CI)	*P* value	HR^*^(95% CI)	*P* value
All-cause Mortality						
PIV	1.0006 (1.0005 ~ 1.0008)	<0.001	1.0003 (1.0002 ~ 1.0005)	<.0001	1.0004 (1.0001 ~ 1.0007)	0.009
PIV (Quartile)						
Q1	1.0000 (Reference)		1.0000 (Reference)		1.0000 (Reference)	
Q2	1.2065 (0.9805 ~ 1.4846)	0.076	1.4759 (1.1935 ~ 1.8252)	<0.001	1.6018 (1.0137 ~ 2.5314)	0.047
Q3	1.7456 (1.4347 ~ 2.1239)	<0.001	1.6628 (1.3574 ~ 2.0368)	<0.001	1.7031 (1.1028 ~ 2.6304)	0.016
Q4	1.9841 (1.6353 ~ 2.4072)	<0.001	1.9204 (1.5700 ~ 2.3490)	<0.001	2.1167 (1.3315 ~ 3.3651)	0.002
*P* for trend	<.0001		<.0001		<.0001	

HR^*^ (Hazard Ratio): A measure of the risk of an event occurring in one group compared to a reference group. An HR > 1 indicates increased risk, while an HR < 1 suggests reduced risk.

Model 1 (Crude): Unadjusted analysis.

Model 2 (Minimally Adjusted): Adjusted for Gender, Race/Ethnicity, Education Level, Marital Status, and Age.

Model 3 (Fully Adjusted): Adjusted for Gender, Race/Ethnicity, Education Level, Smoking, Hypertension, Diabetes Mellitus, Alcohol Consumption, Marital Status, Age, Weight, Standing Height, Waist Circumference, BMI, Triglycerides, LDL-Cholesterol, HDL-Cholesterol, and Total Cholesterol.

In the comparison across PIV quartiles, results showed a significant increase in the risk of all-cause mortality with higher PIV levels. Compared to the first quartile (Q1), the fully adjusted model (Model 3) showed a multivariable-adjusted HR of 1.6018 (95% CI: 1.0137–2.5314, P = 0.0436) for Q2, 1.7031 (95% CI: 1.1028–2.6304, P = 0.0163) for Q3, and 2.1167 (95% CI: 1.3315–3.3651, P = 0.0015) for Q4. The trend test also indicated a statistically significant association across quartiles (P for trend < 0.0001), suggesting that higher PIV levels are closely associated with an increased risk of all-cause mortality in this population.

**Figure 2:**
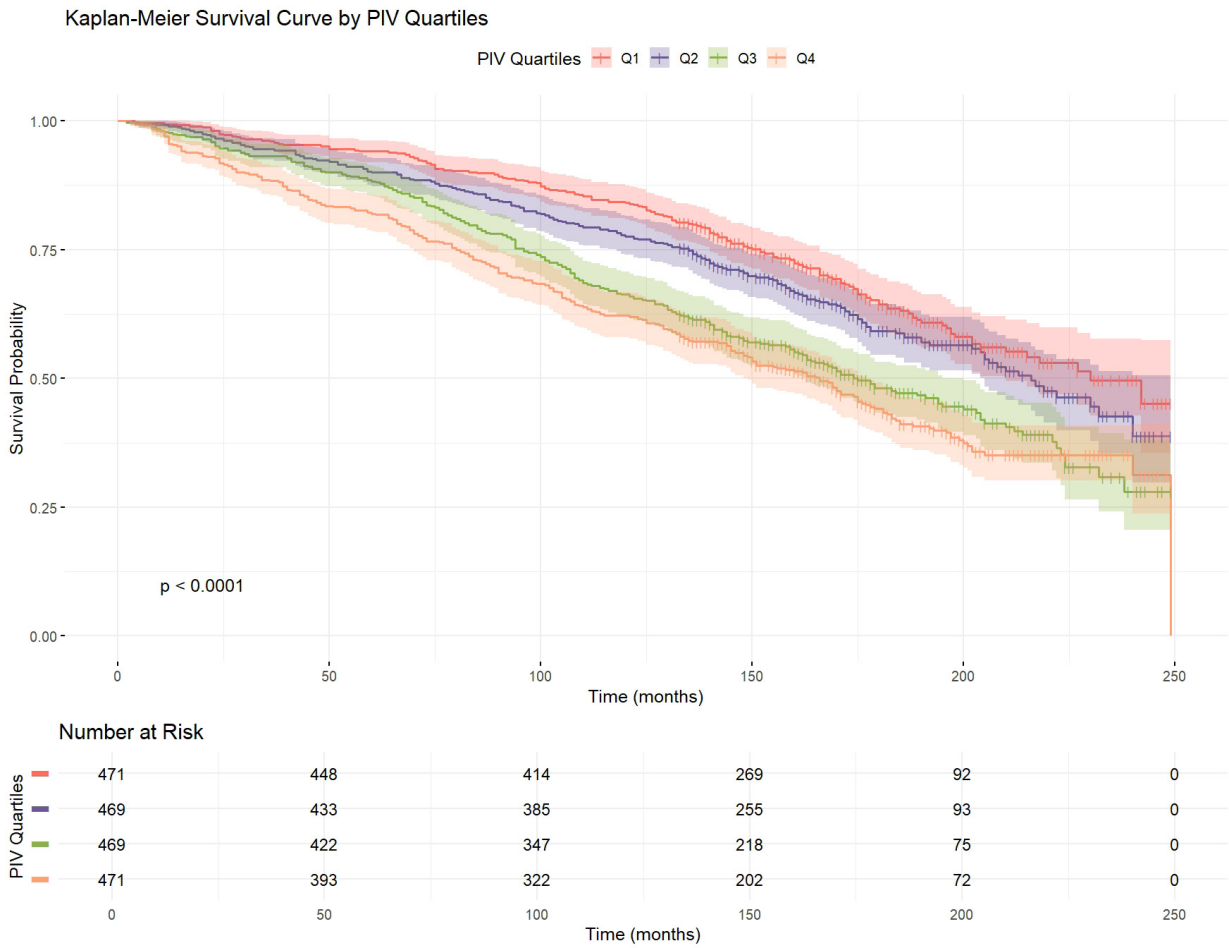
Kaplan-Meier survival curve of RA patients by PIV quartiles.

To further illustrate the association between PIV quartiles and survival probability, Kaplan-Meier survival curves were plotted (**Figure 2**). The survival curves show that higher PIV quartiles were associated with reduced survival probability, highlighting the adverse impact of increased PIV on patient survival. As observed in the curves, the survival rates significantly decreased as the PIV quartile increased (P < 0.0001), consistent with the findings from the Cox proportional hazards models.

### 3.3 Dose-response relationship between PIV and mortality

**Figure 3:**
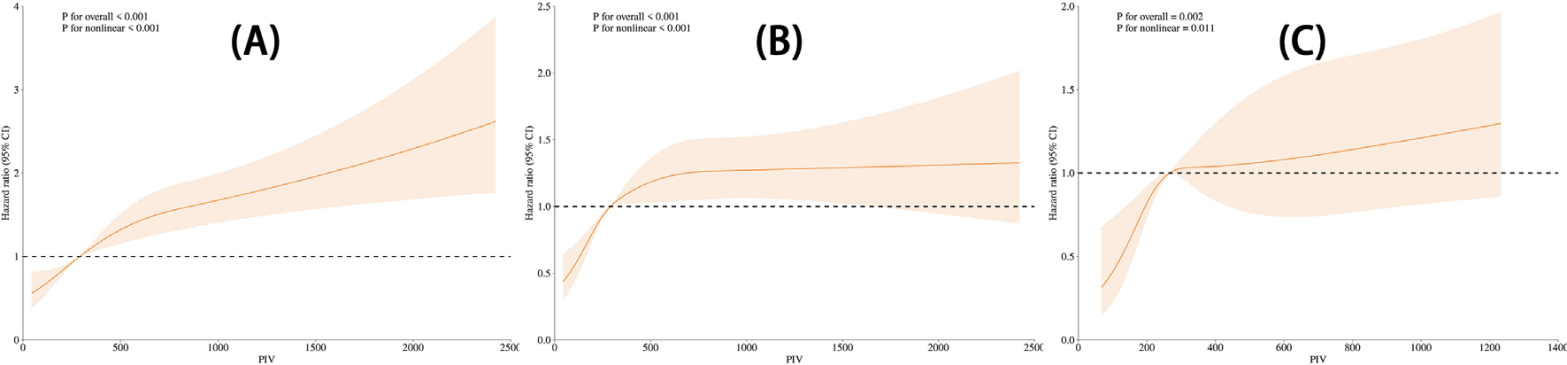
Nonlinear relationship between PIV and mortality across three models. **(A)** Crude Model: Unadjusted. **(B)** Minimally Adjusted Model: Adjusted for gender, race/ethnicity, education level, marital status, and age. **(C)** Fully Adjusted Model: Adjusted for gender, race/ethnicity, education level, marital status, age, smoking, hypertension, diabetes mellitus, alcohol consumption, weight, height, waist circumference, BMI, triglycerides, LDL-cholesterol, HDL-cholesterol, and total cholesterol.

As demonstrated in **Figure 3**, we utilized restricted cubic splines (RCS) based on Cox proportional hazards models to evaluate the nonlinear dose-response relationship between PIV and mortality risk. The analysis was conducted using three models: (A) Crude, (B) Minimally adjusted for demographic variables, and (C) Fully adjusted for a comprehensive set of confounders. In the crude model (A), higher PIV levels were significantly associated with an increased risk of mortality, with a clear upward trend observed throughout the range (P for nonlinear < 0.001). In the minimally adjusted model (B), which controlled for demographic variables such as age, gender, and race, the nonlinear relationship persisted but showed a plateau at higher PIV levels. In the fully adjusted model (C), which accounted for lifestyle factors, metabolic indicators, and chronic conditions, the association remained significant, though the curve indicated a more moderate increase in risk (P for overall = 0.002, P for nonlinear = 0.011). These results confirm the robustness of our approach, demonstrating a consistent dose-response relationship between elevated PIV levels and mortality risk across all models.

### 3.4 Segmented regression analysis

This study employed segmented Cox regression models to explore the nonlinear relationship between the PIV and mortality risk (see **Table 3**). The analysis identified a breakpoint at PIV = 302. Below this threshold (PIV < 302), increasing PIV was significantly associated with a higher mortality risk (HR = 1.6700, 95% CI: 1.0700–2.6100, P = 0.0237). Conversely, above this threshold (PIV ≥ 302), further increases in PIV were linked to a reduced mortality risk (HR = 0.9800, 95% CI: 0.9700–0.9900, P = 0.0263). The log-likelihood ratio test (P < 0.0001) confirmed that the segmented model provided a significantly better fit than the standard Cox regression model. These findings underscore the nonlinear nature of the association between PIV and mortality risk.

**Table 3:** Results of Cox regression and segmented regression analysis of PIV and mortality risk.

Model	Hazard Ratio (HR) Adjusted β (95% CI)	P value
Model 1		
The standard Cox model	1.0004 (1.0001 ~ 1.0007)	0.0085
Model 2		
Turning point (K)	302	
PIV < 302	1.6700 (1.0700 – 2.6100)	0.0237
PIV ≥ 302	0.9800 (0.9700 – 0.9900)	0.0263
Log likelihood ratio test	<0.0001	

Model 1 was a standard Cox regression model that included all covariates, while Model 2 was a segmented Cox regression model incorporating the same set of covariates with a piecewise approach to identify a potential turning point in the relationship between PIV and mortality. The log likelihood ratio test was used to compare the two models, indicating the improved fit of the segmented model.

### 3.5 Subgroup analysis Analysis of the impact Impact of PIV on mortalityMortality

In the subgroup analysis (**Table 4**), the association between PIV and mortality was consistent across most populations, with interaction P-values for age, gender, smoking, alcohol consumption, and hypertension all above 0.05. However, a significant interaction was noted in the diabetes subgroup (P = 0.002). Among non-diabetic patients, higher PIV levels were linked to increased mortality risk (HR = 1.0007, 95% CI: 1.0006–1.0008, P < 0.001), whereas this association was not significant in diabetic patients (HR = 1.0000, 95% CI: 0.9996–1.0005, P = 0.862). These results suggest diabetes may modify the impact of PIV on mortality, emphasizing the need for tailored risk assessments in diabetic patients.

**Table 4:** Subgroup analysis of the impact of PIV on all-cause mortality in rheumatoid arthritis patients.

Variables	n (%)	HR (95%CI)	P	P for interaction
All patients	1882 (100.00%)	1.0006 (1.0005 ~ 1.0008)	<0.001	
Age				0.088
Age≥65	1067 (56.70%)	1.0004 (1.0003 ~ 1.0006)	<0.001	
Age<65	815 (43.30%)	1.0009 (1.0004 ~ 1.0013)	<0.001	
Gender				0.445
Male	675 (35.87%)	1.0007 (1.0004 ~ 1.0009)	<0.001	
Female	1207 (64.13%)	1.0007 (1.0004 ~ 1.0009)	<0.001	
Smoking				0.175
Yes	995 (52.87%)	1.0007 (1.0005 ~ 1.0008)	<0.001	
No	887 (47.13%)	1.0005 (1.0002 ~ 1.0007)	<0.001	
Alcohol Consumption				0.079
Yes	1257 (66.79%)	1.0007 (1.0005 ~ 1.0009)	<0.001	
No	625 (33.21%)	1.0004 (1.0002 ~ 1.0007)	<0.001	
Hypertension				0.417
Yes	1068 (56.75%)	1.0006 (1.0004 ~ 1.0007)	<0.001	
No	814 (43.25%)	1.0007 (1.0004 ~ 1.0010)	<0.001	
Diabetes Mellitus				0.002
Yes	295 (15.67%)	1.0000 (0.9996 ~ 1.0005)	0.862	
No	1587 (84.33%)	1.0007 (1.0006 ~ 1.0008)	<0.001	

HR, Hazard Ratio; CI, Confidence Interval.

## 4 Discussion

### 4.1 Major findings and clinical implications

This study systematically examined the relationship between the PIV and all-cause mortality in patients with RA. The results demonstrated that PIV, as a biomarker reflecting systemic immune-inflammatory activity, is significantly associated with all-cause mortality. Higher PIV levels were linked to an increasing trend in mortality, characterized by a clear dose-response relationship. Multivariable Cox proportional hazards models and segmented regression analysis further highlighted the nonlinear nature of this association. Specifically, a distinct inflection point at a PIV threshold of 302 indicated a sharp increase in mortality risk, reinforcing the potential of PIV as a prognostic tool.

Kaplan-Meier survival curve analysis corroborated these findings, revealing pronounced differences in survival rates across PIV quartiles. Patients in the highest PIV quartile (Q4) exhibited significantly lower survival rates compared to those in the lowest quartile (Q1), underscoring the adverse prognostic implications of elevated PIV levels. Subgroup analyses showed consistent associations between PIV and mortality across various demographic and clinical subgroups, such as age, sex, and smoking status. Although some interaction effects were noted, such as in patients with diabetes, the overall trend remained robust, affirming the broad applicability of PIV as a universal risk assessment metric.

In summary, these findings provide strong evidence supporting the clinical utility of PIV in RA management. PIV offers promise not only as a reliable tool for individualized risk assessment but also as a foundation for optimizing therapeutic strategies, including targeted interventions aimed at improving patient outcomes.

### 4.2 Mechanisms of PIV and its comparison with existing studies

The PIV is a comprehensive inflammatory and immune biomarker derived from complete blood counts by integrating neutrophil, monocyte, platelet, and lymphocyte counts (13, 14). It provides a holistic reflection of systemic immune-inflammatory activity. The key advantage of PIV lies in its ability to consolidate multiple blood parameters, capturing the dynamic variations of inflammatory responses across multiple dimensions (15). This approach addresses the limitations of single markers such as the neutrophil-to-lymphocyte ratio (NLR) or platelet-to-lymphocyte ratio (PLR), offering a more comprehensive assessment of systemic inflammation. Studies have shown that elevated PIV levels are closely associated with the excessive release of inflammatory mediators, immune cell dysregulation, and exacerbation of tissue damage (16–19). These mechanisms are thought to play a critical role in driving the increased all-cause mortality observed in patients with RA.

Yu et al. (2024) conducted a meta-analysis highlighting the substantial prognostic value of PIV in gastrointestinal cancers. Patients with elevated PIV levels were consistently associated with poorer overall survival rates and unfavorable prognoses. These findings suggest that PIV effectively captures the severity of systemic inflammation, serving as a crucial tool for risk stratification in this patient population​ (20). In a study on colorectal cancer, Wang et al. (2024) further validated PIV’s role as a biomarker. Their findings revealed that PIV not only predicts patient survival outcomes but also varies in predictive value depending on the primary tumor location, highlighting its ability to reflect the inflammatory heterogeneity of specific diseases​ (21). Furthermore, in a study on locally advanced nasopharyngeal cancer, Topkan et al. (2024) emphasized the potential of PIV as a prognostic tool before chemoradiotherapy. Their findings revealed that patients with elevated PIV levels exhibited lower disease-free survival rates, underscoring the value of PIV as a dynamic marker for monitoring inflammation during cancer treatment​ (22). Similarly, in patients with advanced pancreatic cancer, Aydin et al. (2024) reported that higher PIV levels were significantly linked to shorter survival times, further reinforcing the broad applicability of PIV as a marker of systemic inflammation​ (23). In the field of cardiovascular diseases, Zhou et al. (2024) examined the association between perioperative changes in PIV and outcomes following cardiovascular surgery. Their findings showed that elevated PIV levels predicted a higher incidence of postoperative complications, further supporting the utility of PIV in diverse disease contexts (24).

This study is the first to validate the utility of PIV in RA, demonstrating its effectiveness as a predictor of all-cause mortality while also highlighting its nonlinear relationship with mortality risk. Segmented regression analysis identified a specific threshold (PIV = 302) at which a notable inflection point in mortality risk occurred, underscoring PIV’s sensitivity in reflecting the dynamic interplay of inflammation and immune dysregulation. Compared to existing literature, this study introduces two key innovations. First, it extends the application of PIV beyond acute conditions (e.g., cancer) to chronic inflammatory diseases like RA, providing a theoretical foundation for its broader use in systemic inflammatory disorders. Second, by employing advanced statistical methods such as segmented regression analysis, it offers a more comprehensive understanding of the intricate relationship between PIV and mortality, paving the way for future research on dynamic inflammatory biomarkers. These findings not only deepen our understanding of the mechanisms underlying PIV but also establish a strong foundation for its potential role in the management of RA patients, particularly in personalized risk assessment and targeted therapeutic interventions.

### 4.3 Clinical implications and future research directions

This study underscores the clinical potential of the PIV as a risk assessment tool by demonstrating its nonlinear association with all-cause mortality in patients with RA. These findings hold significant implications for both clinical practice and future research.

In clinical settings, PIV stands out as a low-cost, highly accessible inflammatory biomarker with broad potential applications. Derived from routine complete blood counts, it is adaptable to various healthcare environments, including resource-limited settings. Compared to traditional inflammatory markers such as C-reactive protein (CRP), the neutrophil-to-lymphocyte ratio (NLR), or the platelet-to-lymphocyte ratio (PLR), PIV integrates multiple inflammatory and immune parameters, offering a more holistic representation of systemic inflammation. This allows clinicians to achieve more precise patient risk stratification.

PIV is particularly valuable in identifying high-risk RA patients, especially those with a heightened risk of all-cause mortality. Identifying these individuals enables clinicians to refine treatment strategies, such as employing more aggressive anti-inflammatory and immunomodulatory therapies while closely monitoring disease progression. Moreover, PIV serves as a dynamic tool for tracking treatment responses. Evidence suggests that fluctuations in PIV levels closely correspond with changes in inflammatory status, making it an effective marker for real-time assessment of therapeutic outcomes and facilitating timely interventions.

On the research front, further studies are essential to validate and optimize the use of PIV. First, researchers should evaluate its generalizability across diverse populations, including variations in ethnicity, sex, age, and comorbidities. Given the heterogeneity of inflammatory responses across different diseases, large-scale, multicenter studies are crucial to confirm PIV’s global applicability. Second, future investigations should explore the temporal dynamics of PIV. While most existing studies focus on baseline measurements, longitudinal tracking of PIV could provide deeper insights into its relationship with inflammatory states and disease risks. Such studies would clarify PIV’s role in disease progression and treatment monitoring.

Additionally, integrating PIV with other inflammatory or metabolic markers—such as CRP, serum ferritin, or indicators of metabolic syndrome—could enhance its predictive accuracy and strengthen its utility in personalized medicine. Finally, the biological mechanisms underlying PIV’s relationship with clinical outcomes remain insufficiently understood. Current evidence indicates that PIV reflects systemic inflammation, immune dysregulation, and tissue damage; however, its precise pathways remain unclear. Advanced approaches leveraging genomic, proteomic, and metabolomic analyses could elucidate these mechanisms and guide the development of novel anti-inflammatory treatments.

In conclusion, PIV has significant potential for clinical applications. However, further research is needed to assess its performance in diverse populations, its dynamic behavior over time, and its combined use with other biomarkers. These efforts will advance PIV’s integration into personalized medicine, ultimately improving prognostic accuracy and health outcomes for RA patients.

### 4.4 Strengths and limitations of the research methods

This study leveraged a large, nationally representative dataset, alongside multivariable Cox proportional hazards models and segmented regression analysis, to uncover the nonlinear relationship between the PIV and all-cause mortality in patients with RA. Kaplan-Meier survival curve analyses and subgroup evaluations further validated the robustness of the findings and explored variations within specific populations, providing preliminary support for the individualized application of PIV.

Despite these strengths, the study has several limitations that should be addressed in future research. First, as an observational study, it cannot definitively establish causality. While extensive multivariable adjustments were performed to minimize confounding, unmeasured variables or residual confounding may still influence the results. For instance, certain inflammation-related variables or patient-specific factors not included in the model could play a significant role.

Second, PIV was calculated from a single blood test, which may not fully capture long-term inflammatory dynamics. Inflammatory responses can vary significantly over time, and single measurements may fail to reflect these fluctuations. Future longitudinal studies incorporating repeated PIV measurements could better assess how temporal variations impact patient outcomes. Furthermore, this study did not combine PIV with other inflammatory biomarkers, such as C-reactive protein (CRP) or serum ferritin, which could limit the comprehensiveness of the findings. A combined approach may enhance PIV’s predictive power and provide deeper insights for clinical application.

Third, the study was conducted within a specific geographic region, and the findings may not generalize to other populations or healthcare systems. Immune and inflammatory patterns can vary widely across ethnicities, which might influence the utility of PIV in global contexts. To address this, multicenter and multiethnic studies are needed to confirm the generalizability and stability of PIV as a biomarker.

Finally, the study did not explore the underlying biological mechanisms of PIV in depth. While the association of PIV with systemic inflammation and immune dysregulation is well-supported, its precise molecular and biological pathways remain unclear. Future research could employ multi-omics approaches, such as genomics, proteomics, and metabolomics, to investigate the connections between PIV and specific inflammatory pathways, shedding light on its role as an inflammatory biomarker.

In summary, this study rigorously demonstrated the nonlinear relationship between PIV and all-cause mortality in RA patients, establishing its potential as a prognostic tool. However, addressing these limitations—such as validating causality, assessing dynamic changes, exploring global applicability, and investigating underlying mechanisms—will be crucial for advancing the clinical utility of PIV and enhancing its impact on patient care.

## 5 Conclusion

This study systematically examined the association between the PIV and all-cause mortality in patients with RA, revealing a significant nonlinear relationship. Through multivariable Cox regression and segmented regression analyses, a critical PIV threshold (302) was identified, at which mortality risk sharply increased. These findings underscore the potential of PIV as a prognostic tool, not only for predicting all-cause mortality but also for facilitating personalized risk assessment based on robust data. Furthermore, this study validates the applicability of PIV in chronic inflammatory diseases, establishing a foundation for future research into its generalizability across diverse populations and its integration with other biomarkers. Moving forward, PIV shows great promise in advancing precision medicine for RA and other systemic inflammatory diseases, offering vital support for optimizing treatment strategies and improving patient outcomes.

## Data Availability

Publicly available datasets were analyzed in this study. This data can be found here: https://www.cdc.gov/nchs/nhanes/?CDC_AAref_Val=https://www.cdc.gov/nchs/nhanes/index.htm.
